# Engeletin protects against cerebral ischemia/reperfusion injury by modulating the VEGF/vasohibin and Ang-1/Tie-2 pathways

**DOI:** 10.1590/1414-431X2020e11028

**Published:** 2021-07-16

**Authors:** Hui Liu, Shucui Li, Yangyang Xu, Xin Wang, Rui Ren, Haibo Zhu, Shuping Zhang

**Affiliations:** 1 Department of Pharmacology, Binzhou Medical University, Yantai, Shandong, China; 2 School of Public Health and Management, Binzhou Medical University, Yantai, Shandong, China; 3 Department of Pharmacy, Binzhou Medical University Hospital, Binzhou, Shandong, China

**Keywords:** Engeletin, Angiogenesis, Oxygen-glucose deprivation reperfusion, Cerebral ischemia-reperfusion, VEGF/vasohibin pathway, Ang-1/Tie-2 pathway

## Abstract

Engeletin is a natural derivative of *Smilax glabra rhizomilax* that exhibits anti-inflammatory activity and suppresses lipid peroxidation. In the present study, we sought to elucidate the mechanistic basis for the neuroprotective and pro-angiogenic activity of engeltin in a human umbilical vein endothelial cells (HUVECs) oxygen-glucose deprivation and reoxygenation (OGD/R) model system and a middle cerebral artery occlusion (MCAO) rat model of cerebral ischemia and reperfusion injury. These analyses revealed that engeletin (10, 20, or 40 mg/kg) was able to reduce the infarct volume, increase cerebral blood flow, improve neurological function, and bolster the expression of vascular endothelial growth factor (VEGF), vasohibin-2 (Vash-2), angiopoietin-1 (Ang-1), phosphorylated human angiopoietin receptor tyrosine kinase 2 (*p*-Tie2), and platelet endothelial cell adhesion molecule-1 (PECAM-1/CD31) in MCAO rats. Similarly, engeletin (100, 200, or 400 nM) markedly enhanced the migration, tube formation, and VEGF expression of HUVECs in an OGD/R model system, while the VEGF receptor (R) inhibitor axitinib reversed the observed changes in HUVEC tube formation activity and Vash-2, VEGF, and CD31 expression. These data suggested that engeletin exhibited significant neuroprotective effects against cerebral ischemia and reperfusion injury in rats, and improved cerebrovascular angiogenesis by modulating the VEGF/vasohibin and Ang-1/Tie-2 pathways.

## Introduction

Strokes are highly debilitating cerebrovascular and cardiovascular events that are occurring with increasing frequency (1). Approximately 85% of these events are ischemic strokes that arise as a consequence of vascular occlusion (2). Stroke patients are typically treated using a combination of endovascular, thrombolytic, and adjuvant approaches (3,4). However, intravenous tissue plasminogen activator administration is only effective within 4.5 h following stroke (5), and as such, relatively few patients are eligible to undergo this treatment (6). It is therefore essential that the mechanistic basis for cerebral ischemia/reperfusion (CI/R) injury be better understood in order to protect against permanent disability or death in affected patients. Multiple studies have determined that promoting vascular remodeling is an effective means of restoring the blood supply in this pathogenic context (7), highlighting this as a promising therapeutic modality.

Vascular endothelial growth factor (VEGF) (8) is a key mediator of angiogenesis (9), promoting processes such as the proliferation, survival, growth, and migration of endothelial cells (10). VEGF-treated endothelial cells produce vasohibin-1 (Vash-1) (11), which heterodimerizes with small vasohibin-binding protein (SVBP) within cells to drive Vash-1 secretion and the consequent negative feedback for angiogenesis (12). In contrast, CD11b-positive bone marrow-derived mononuclear cells and cancer cells express the homologous Vash-2 protein, which stimulates angiogenic processes (13). Angiopoietin-1 (Ang-1) is a pericyte-derived protein that drives vascular remodeling and angiogenesis by signaling through its cognate receptor tyrosine kinase 2 (Tie-2) on endothelial cells (14). Through these mechanisms, the VEGF/vasohibin pathway controls angiogenesis while the Ang-1/Tie-2 pathway modulates the permeability and stability of the newly formed vasculature.

Engeletin, which is also referred to as rhamnoside of dihydrokaempferol, is a bioactive derivative of *Smilax glabra rhizomilax*. In prior research, engeletin has shown anti-inflammatory and antioxidant activity *in vitro* (15) and ability to further suppress the growth of cervical and lung cancer cells (16,17). Herein, we explored the protective activity of engeletin in the context of CI/R injury and assessed the underlying mechanisms whereby it regulates angiogenesis.

## Material and Methods

### Drugs and reagents

Engeletin (>99.0% pure, molecular weight: 434.39, CAS: 572-31-6) was obtained from SenBeiJia Biological Technology (China) and was dissolved using dimethyl sulfoxide (DMSO) to prepare a stock solution.

Axitinib (a VEGF receptor (R) inhibitor, Cat No. SC0137-25 mg) and anti-VEGF (Cat No. AV202) were purchased from Beyotime Biotechnology (China). Anti-vasohibin-1 antibody (Cat No.ab176114), anti-Ang-2 (Cat No. ab180820), and anti-Ang-1 (Cat No. ab102015) were from Abcam (UK). Anti-*p*-Tie2 (Cat No. SAB4503999) and anti-NeuN (Cat No. MEB377) were from Sigma (China). Vasohibin-2 (Cat No. 2923051) was obtained from EMD Millipore (Germany). HRP-labeled goat anti-rabbit IgG (Cat No. A0208), HRP-labeled goat anti-mouse IgG (Cat No. A0216), mouse anti-actin (Cat No. AA128), and BeyoECL Plus (Cat No. P0018FS) were from Beyotime Biotechnology. The 4-0 filament (filament diameter: 0.25 mm; tip diameter: 0.34 mm) was purchased from Beijing Shadong Biology Company (China). RPMI-1640 (Cat No. 11875-093) was from Gibco (USA). Fetal bovine serum (FBS; Cat No.13011-8611) was from Sijiqing (China).

### Cell culture

HUVECs (ATCC, USA) were cultured in RPMI-1640 containing 10% FBS and penicillin/streptomycin at 37°C in a humidified 5% CO_2_ incubator.

### MCAO model establishment

Male Sprague-Dawley rats (220-250 g) were purchased from Jinan Pengyue (China) Experimental Animal Breeding Co., Ltd. (SPF grade, Certificate No. SYXK 20170018) and housed with access to food and water *ad libitum* for 7 days to facilitate acclimatization. Rats were then anesthetized via intramuscular injection of ketamine (100 mg/kg) and xylazine (10 mg/kg), after which the middle cerebral artery occlusion (MCAO) procedure was conducted via a slightly modified version of previously published protocols (18). Briefly, the left common carotid artery was blocked, while the external carotid artery branches were divided following dissection, and a 4-0 filament was inserted into the internal carotid artery until resistance was detected. After 1.5 h, this filament was removed to facilitate reperfusion. For 24 h following surgery, rats were maintained at 24-25°C. The Guide for the Care and Use of Laboratory Animals (NIH Publications No. 80-23, USA), revised in 1996, was followed when conducting all animal studies. All experiments and procedures were conducted with the approval of the Animal Care Guidelines of the Animal Experiment Committee of Binzhou Medical University (China; authorization number BYLY 2019-056).

### Animal treatment groups

For dose-response studies, 60 rats were randomly assigned to 5 different treatment groups (12 per group): a sham group, an MCAO group, and three engeletin treatment groups (10, 20, and 40 mg/kg). At 15 min after reperfusion, appropriate engeletin doses were intraperitoneally administered to the indicated treatment groups, with sham and MCAO model animals being administered normal saline. At 24-h post-modeling, neurological function assays were performed. Rats were then anesthetized using ketamine and xylazine as above, and the optimal engeletin treatment dose was established by analyzing survival rates, brain water content, and infarct area (as assessed via 2,3,5-triphenyl-tetrazolium-chloride [TTC] staining).

For mechanistic studies, 50 rats were randomized into five treatment groups (10 per group): a sham group, an MCAO group, an MCAO + engeletin (20 mg/kg *ip*) group, an MCAO+axitinib (10 mg/kg, *ig*) group, and an MCAO+engeletin+axitinib group. Engeletin and axitinib were administered continuously for 7 days. Cerebral blood flow was measured prior to MCAO and at 0, 72, and 168 h post-MCAO. On day 8, rats were euthanized and the cortical tissue around the ischemic penumbra was collected for immunohistochemical and western blotting analyses. Sham and MCAO model rats were administered appropriate volumes of normal saline in lieu of treatment.

### Cerebral blood flow measurements

Cerebral blood flow was measured via moorFLPI-2 (Moor Instruments, UK), with measurements being made before MCAO modeling and at 0, 72, and 168 h post-engeletin treatment. Following blood flow measurements, engeletin or normal saline were administered as appropriate. Real-time flux data were obtained and analyzed with the moorFLPI-2 measurement software.

### Neurological function tests

The modified neurological severity score in rats (mNSS) was assessed by a researcher blinded to experimental grouping (19). Briefly, neurological function was graded from 0 (normal) to 18 (severe neurological damage). Overall mNSS scores were assigned based upon a composite of reflex, balance, and sensory testing results, with one point being awarded for each task that an animal was unable to perform or each tested reflex that was absent.

### Immunohistochemical analyses

Immunohistochemical staining was conducted as in prior reports (20). Briefly, 4% paraformaldehyde (PFA) was used to fix brain tissues overnight before paraffin embedding. Next, serial 4-μm thick cerebral cortex sections were prepared, deparaffinized, rehydrated, and subjected to 0.01 M citric acid treatment for 10 min in a microwave at 400 W. Sections were treated for 12 min to block endogenous peroxidase activity, blocked for 30 min with bovine serum albumin (BSA), and treated overnight with polyclonal antibodies specific for VEGF (Beyotime Biotechnology, 1:200), CD31 (Abcam, 1:50), and NeuN (Sigma, 1:100) at 4°C. Sections were then washed and probed using an HRP-conjugated secondary antibody (Beyotime Biotechnology) for 1 h at 37°C, after which samples were evaluated via light microscopy (Perkin Elmer, USA). Integrated absorbance values were calculated by Vectra 3 (Akoya Biosciences, USA) to determine the positive protein expression area as follows: integrated absorbance = positive area × average absorbance.

### OGD/R model establishment

An *in vitro* oxygen-glucose deprivation and reoxygenation **(**OGD/R) model was established as in prior studies (21). Briefly, HUVECs were treated under hypoxic conditions for 4 h in Earle's solution that had been treated with N_2_ for 30 min prior to use. Hypoxia was achieved with a hypoxic incubator (Kendro; Thermo Fisher Scientific, Inc., USA), with respective O_2_ and CO_2_ levels of 1.0 and 5.0%. At 4 h post-OGD, reoxygenation was conducted by exchanging this medium for standard serum-free culture media containing engeletin (0, 50, 100, 200, or 400 nM) in a normoxic incubator.

### Cell proliferation assay

A CCK-8 assay was employed to test HUVEC viability based on manufacturer’s instructions (Beyotime Biotechnology). Briefly, HUVECs were added onto 96-well plates (6,000/well) for 24 h. Following OGD/R, cells were incubated with appropriate engeletin concentrations for 24 h, after which CCK-8 solution was added (10 μL/well) for 4 h at 37°C. Absorbance at 450 nm was then assessed with a Molecular Devices SpectraMax M 5 instrument (USA).

Proliferation was also assessed via flow cytometry. First, HUVECs were plated at 25×10^4^ cells/well onto 6-well plates, followed by OGD/R and engeletin treatment as above. Cells were then harvested, fixed, stained, and assessed with a flow cytometer (BC Biosciences, USA) based upon PI staining results according to provided protocols.

### Transwell assay

The impact of engeletin on cellular migration was assessed using 12-well Transwell assay inserts (3462, Corning, USA) as *in priori* reports (22). Briefly, HUVECs were resuspended in serum-free medium with engeletin (50, 100, 200, or 400 nM) in the upper chamber, while the lower chamber was filled with standard medium. Following a 24-h incubation, cells in the upper chamber were carefully removed with a swab, while the remaining cells were fixed and stained with crystal violet. Cells in three random fields of view per well were then counted via an inverted microscope (Olympus, Japan).

### Wound healing assay

Cell migration was also evaluated via wound healing assay. Briefly, HUVECs were grown overnight to confluence in 6-well plates, after which a sterile 200-μL pipette was used to generate a scratch wound in the monolayer and PBS was used to wash the cells two times. Engeletin (50, 100, 200, or 400 nM) was then applied, and three random fields of view per image were analyzed at 0 and 24 h, with Photoshop software (Microsoft, USA) being used to quantify the degree of migration.

### Tube formation assay

Capillary-like tube formation by HUVECs in Matrigel was used to measure the neovascularization-like activity of engeletin as detailed previously (23). Briefly, 24-well plates were coated for 30 min using Matrigel (180 μL; 356234, Corning) at 37°C. After polymerization was complete, cells were suspended in RPMI-1640 and applied to the Matrigel at 6×10^4^ cells/well with appropriate engeletin concentrations (0, 50, 100, 200, or 400 nM) and axitinib (1 μM) for 6 h. An inverted microscope was then used to visualize tube structures, which were manually counted.

### Western blotting

Western blotting was used to measure protein levels in cortical or cellular samples as discussed previously (24). Briefly, samples were lysed in RIPA buffer and centrifuged at 13,362 *g* for 15 min at 4°C. Protein samples (40 µg) were then separated via 10% SDS-PAGE and transferred to PVDF membranes that were blocked overnight with antibodies specific for VEGF, Vash-1, Vash-2, Ang-1, Ang-2, or *p*-Tie2. Blots were then washed, probed with secondary antibodies, and protein bands were visualized with BeyoECL Plus and an enhanced chemiluminescence system (Sage Creation, China).

### Statistical analysis

Data were first tested for normal distribution. Non-normally distributed data are reported as medians and interquartile range, and were analyzed using Kruskal-Wallis non-parametric test. Normally distributed data are reported as means±SD, and Levene's test of data homogeneity was performed. If the data were homogeneous (P>0.05), one-way ANOVA was performed followed by Tukey's test. If the Levene's test result was significant (P≤0.05), the Kruskal-Wallis nonparametric test was performed. All the analyses were conducted using SPSS 22.0 (IBM, USA).

## Results

### Effects of engeletin on infarct volume, brain water content, and neurological function

We began by exploring the dose-dependent impact of engeletin in the MCAO model system. At 24-h post-MCAO, 3, 2, 1, and 0 rats in the MCAO, 10, 20, and 40 mg/kg engeletin groups had died, respectively. TTC staining was employed to assess the infarct volume in these rats, with ischemic and non-ischemic regions appearing white and red, respectively. The infarct volume in the MCAO group at 24 h post-CI/R was 23%, and decreased in a dose-dependent manner in the engeletin treatment groups such that it was significantly lower in rats treated with 20 and 40 mg/kg engeletin (F=7.281, P<0.05 and P<0.01) (Table 1). To explore the functional impact of such engeletin treatment, we additionally assessed rat neurological function via mNSS testing and found that the functional recovery of rats in all three engeletin treatment groups was significantly better than that of rats in the MCAO group (F=15.282, P<0.05 or P<0.01) (Table 1), with engeletin functioning in a dose-dependent manner. As such, a 20 mg/kg engeletin dose was selected for subsequent mechanistic studies.

**Table 1:** The dose-effect relationship of engeletin on cerebral ischemia/reperfusion injury in rats.

Group	Survival rate(%)	Infarct volume(%)	Brain water content(%)	Neurological function (score)
Sham	100	-	76.1±0.5	-
MCAO	66.66	22.3±5.7	78.1±0.5^##^	9.4±1.5
Engeletin, 10 mg/kg	75	20.1±5.4	77.4±0.4	7.7±1.5*
Engeletin, 20 mg/kg	75	18.3±4.7*	77.3±0.4*	7.4±1.6**
Engeletin, 40 mg/kg	88.33	17.2±4.1**	77.0±0.5**	7.2±1.7**

Data are reported as means±SD (n=12/group). *P<0.05, **P<0.01 *vs* middle cerebral artery occlusion (MCAO) group; ^##^P<0.01 *vs* sham group (ANOVA with Tukey's test).

### Engeletin increased cerebral blood flow following MCAO

moorFLPI-2 was used to assess cerebral blood flow prior to MCAO and at 0, 72, and 168 h post-MCAO, revealing that the blood flow in the ischemic area was markedly decreased in MCAO model animals relative to sham controls. This blood flow increased slightly at 72 and 168 h post-MCAO relative to 0 h post-MCAO, but remained significantly higher in rats treated with engeletin (20 mg/kg; P<0.05) at 72 h (F=24.847) and 168 h (F=21.714) post-MCAO relative to untreated MCAO model group animals (Table 2). However, the administration of the VEGFR inhibitor axitinib (10 mg/kg) was sufficient to abolish this engeletin-dependent restoration of cerebral blood flow (F=35.429, P<0.05; Table 2).

**Table 2:** The impact of engeletin on mean cerebral blood flow in cerebral ischemia/reperfusion model rats.

Group	Before MCAO	MCAO (0 h)	72 h	168 h
Sham	1123.5±150.7	1033.6±134.5	1145.6±169.3	1131.8±201.3
Model	1131.9±127.9	505.6±88.4**	870.5±325.8**	820.4±213.4**
Engeletin, 20 mg/kg	1128.6±122.6	523.66±94.2**	1040.7±342.1^##^	950.4±265.1^##^
Axitinib, 10 mg/kg	1178.6±164.6	513.45±85.7**	733.6±257.4**	785.4±112.3**
Axitinib, 10 mg/kg + engeletin 20 mg/kg	1149.3±167.4	510.48±92.5**	893.6±311.6*	860.5±133.2*

Data are reported as means±SD (n=10/group). *P<0.05, **P<0.01 *vs* sham group; ^##^P<0.01 *vs* Model group (ANOVA with Tukey's test).

### Engeletin enhanced the expression of VEGF, CD31, Ang1, *p*-Tie2, and NeuN

**Figure 1:**
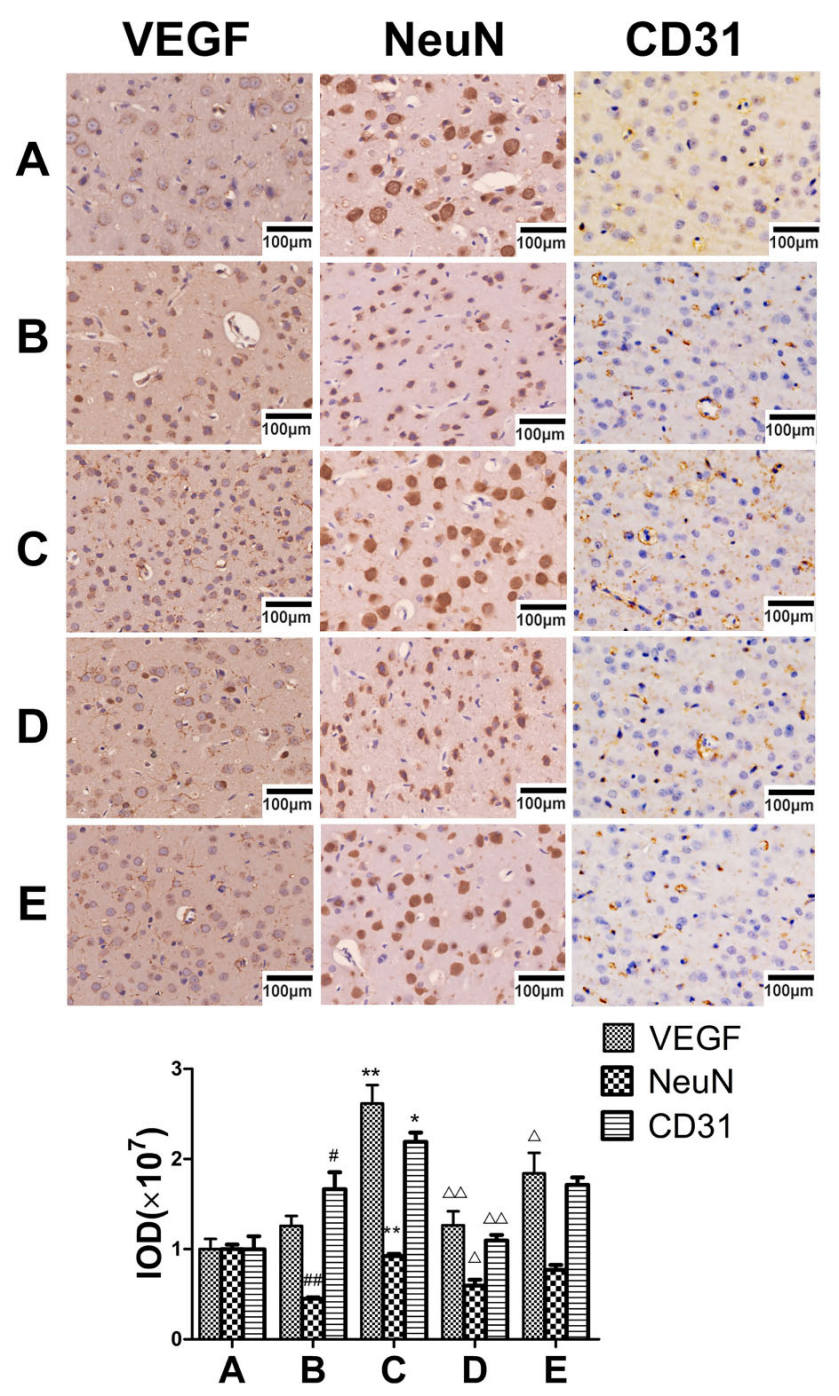
Engeletin protects neurons and promotes cortical vascular endothelial growth factor (VEGF) expression *in vivo*. **A**, sham group. **B**, middle cerebral artery occlusion (MCAO) group. **C**, MCAO + engeletin (20 mg/kg) group. **D**, MCAO+axitinib (10 mg/kg) group. **E**, MCAO + engeletin + axitinib group (scale bar 100 μm). Representative immunohistochemical images following engeletin treatment (20 mg/kg) and axitinib (10 mg/kg). VEGF, NeuN, and CD31 tissue positivity was quantified with the ImageJ software. Data are reported as means±SD (n=10). ^#^P<0.05, ^##^P<0.01 *vs* sham group; **P<0.01 *vs* MCAO group; ^△^P<0.05, ^△△^P<0.01 *vs* engeletin group (ANOVA with Tukey's test).

**Figure 2:**
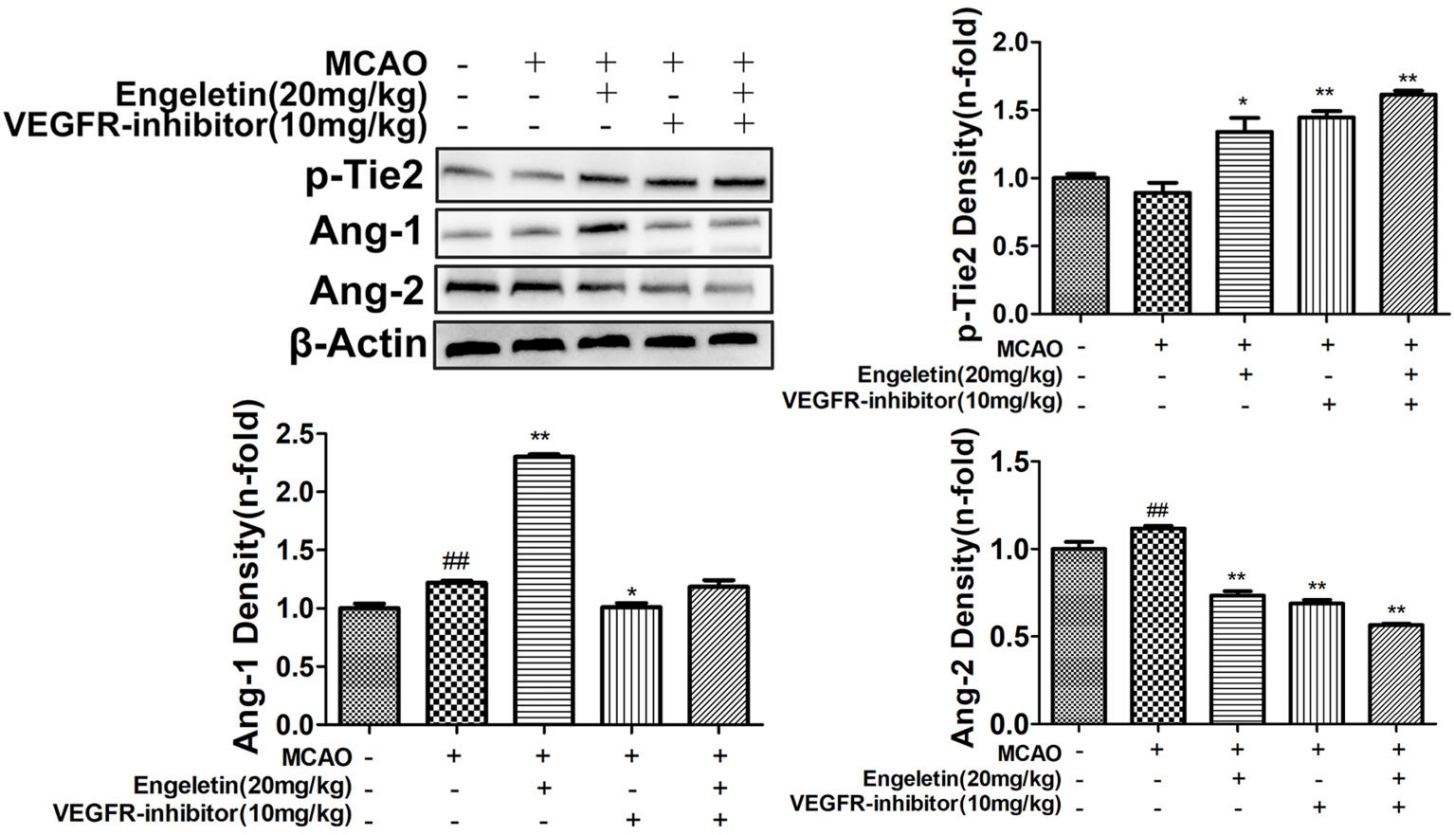
The impact of engeletin on middle cerebral artery occlusion (MCAO)-induced cerebral ischemia/reperfusion injury is mediated via VEGF/vasohibin and Ang-1/Tie-2 signaling. Rats were intragastrically administered axitinib (10 mg/kg, VEGFR-inhibitor) and injected with engeletin (20 mg/kg) following MCAO modeling. Plus and minus signs indicate that a given operation was or was not conducted. Western blotting was used to assess the expression of proteins associated with vascular stability (*p*-Tie2, Ang-1, and Ang-2). Data are reported as means±SD (n=10). *P<0.05, **P<0.01 *vs* MCAO group; ^##^P<0.01 *vs* sham group (ANOVA with Tukey's test).

MCAO model animals exhibited slight increases in VEGF expression relative to sham controls (Figure 1), whereas these levels were significantly elevated following engeletin (20 mg/kg) treatment (F=33.582, P<0.01). The expression of CD31, which is an angiogenesis marker, was also enhanced (F=6.050, P<0.01) in engeletin-treated rats relative to MCAO model rats (Figure 1), suggesting a rise in the number of vessels and a consequent increase in blood flow within the ischemic area. When neurons were examined in MCAO model animals, many appeared shrunken and damaged, consistent with potential neurological damage. In contrast, the morphology of these neurons was restored when treated with engeletin (F=28.317, P<0.01). Similarly, Ang-1 and *p*-Tie2 expression were markedly enhanced in engeletin-treated animals relative to MCAO model animals (Figure 2). Treatment with axitinib (10 mg/kg) was sufficient to suppress the beneficial effects of engeletin, as axitinib treatment was associated with significantly decreased VEGF, Ang-1, *p*-Tie2, NeuN, and CD31 expression relative to engeletin treatment alone (Figures 1 and 2).

### Engeletin promoted HUVEC proliferation following OGD/R

**Figure 3:**
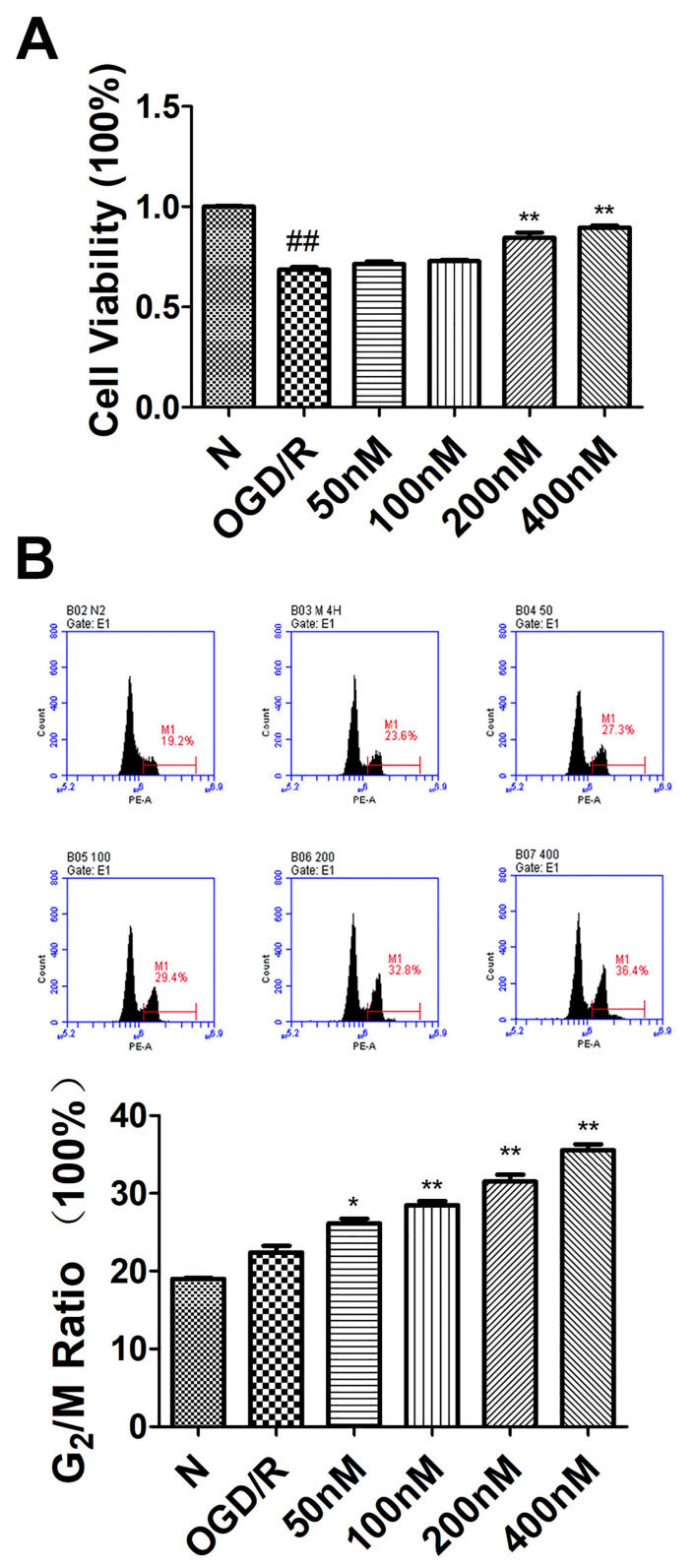
Engeletin enhances human umbilical vein endothelial cell (HUVEC) proliferation in an oxygen-glucose deprivation and reoxygenation (OGD/R) model system. **A**, HUVEC viability following treatment with a range of engeletin concentrations was assessed via CCK-8 assay. **B**, Flow cytometry was used to assess HUVEC cycle progression, with the second peak corresponding to numbers of cells in the G_2_/M phase. Data are reported as means±SD (n=3). *P<0.05, **P<0.01 *vs* the OGD/R group; ^#^P<0.05, ^##^P<0.01 *vs* the normal (N) group (ANOVA with Tukey's test).

A CCK-8 assay revealed that OGD/R treatment markedly impaired the viability of HUVECs, whereas engeletin treatment (200 and 400 nM) was sufficient to enhance the viability of these cells (F=34.165, P<0.01; Figure 3A). To further explore the protective properties of engeletin, cell cycle progression was also assessed, revealing a significant increase (F=45.528, P<0.01) in the ratio of G_2_/M phase cells following engeletin treatment (100-400 nM; Figure 3B). These findings supported a role for engeletin as being protective against OGD/R-induced damage in HUVECS.

### Engeletin enhanced HUVEC migration and tube formation

**Figure 4:**
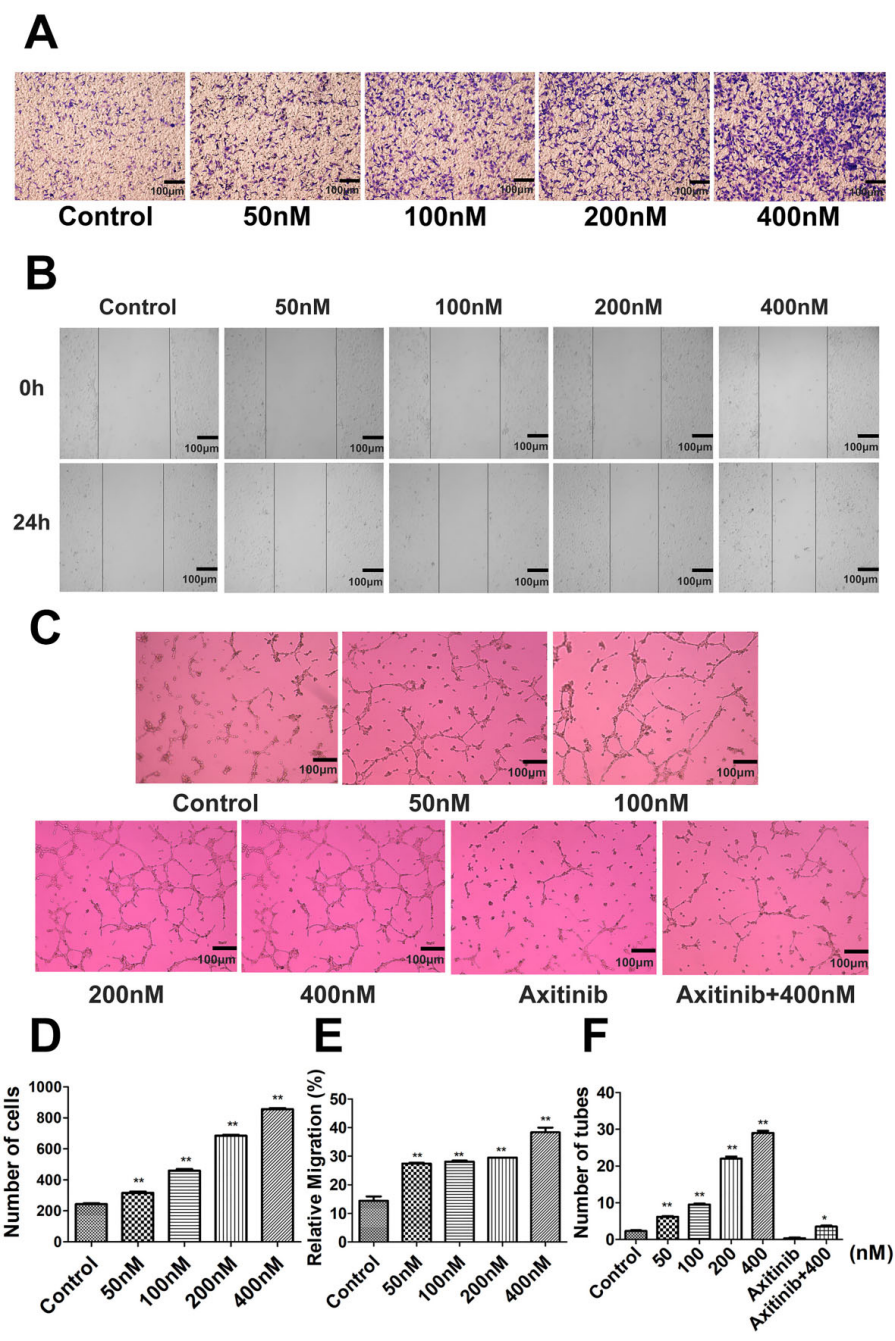
**A** and **B**, human umbilical vein endothelial cell (HUVEC) migration after treatment for 24 h with engeletin (50, 100, 200, 400 nM) was assessed via Transwell or wound healing assay. HUVEC tube formation was assessed via tube formation assay. **C**, Effects of engeletin and axitinib (1 μM) on HUVEC tube formation (scale bar 100 μm). **D**, Transwell assay migration cell count. **E**, Relative migration in the wound healing assay. **F**, Number of tubes in the tube formation assay. *P<0.05, **P<0.01 *vs* control group (ANOVA with Tukey's test).

Endothelial cell migration regulates angiogenesis, and as such, we performed transwell and wound healing assays to explore the impact of engeletin on HUVEC migration. Engeletin treatment (50-400 nM) was sufficient to enhance HUVEC migration in a Transwell assay in a dose-dependent manner relative to control treatment (F=17.136, P<0.01; Figure 4A and D). A wound healing assay yielded similar results (Figure 4B and E), with engeletin markedly enhancing healing relative to control treatment (F=27.728, P<0.01).

We also assessed the angiogenic activity of engeletin by comparing HUVEC morphologic differentiation into capillary-like structures in a Matrigel-based assay. While very few tubular structures were observed in untreated wells (Figure 4C and F), engeletin markedly enhanced the formation of these structures in a dose-dependent manner (F=77.345, P<0.05 or P<0.01). Notably, axitinib (1 μM) co-treatment was sufficient to inhibit the enhanced tube formation mediated by engeletin (F=156.606, P<0.05).

### Effects of engeletin on the VEGF/vasohibin and Ang-1/Tie-2 pathways *in vivo* and *in vitro*

**Figure 5:**
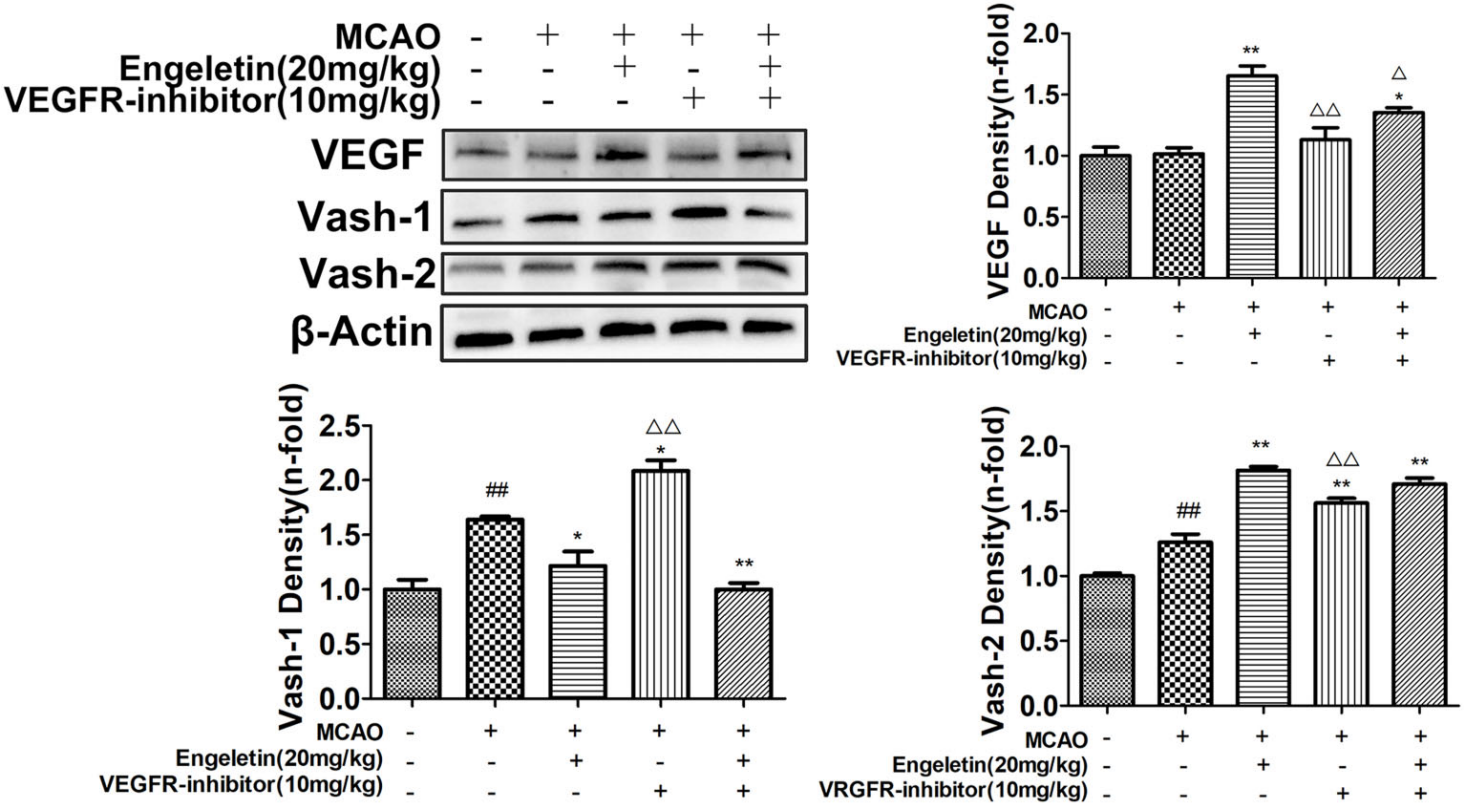
The impact of engeletin on middle cerebral artery occlusion (MCAO)-induced cerebral ischemia/reperfusion injury is mediated via VEGF/vasohibin (Vash) and Ang-1/Tie-2 signaling. Rats were intragastrically administered axitinib (10 mg/kg, VEGFR-inhibitor) and injected with engeletin (20 mg/kg) following middle cerebral artery occlusion (MCAO) modeling. Plus and minus signs indicate that a given operation was or was not conducted. Western blotting was used to assess the expression of proteins associated with angiogenesis (VEGF, Vash-1, and Vash-2). Data are reported as means±SD (n=10). *P<0.05, **P<0.01 *vs* MCAO group; ^##^P<0.01 *vs* sham group. ^△^P<0.05, ^△△^P<0.01 *vs* engeletin group (ANOVA with Tukey's test).

**Figure 6:**
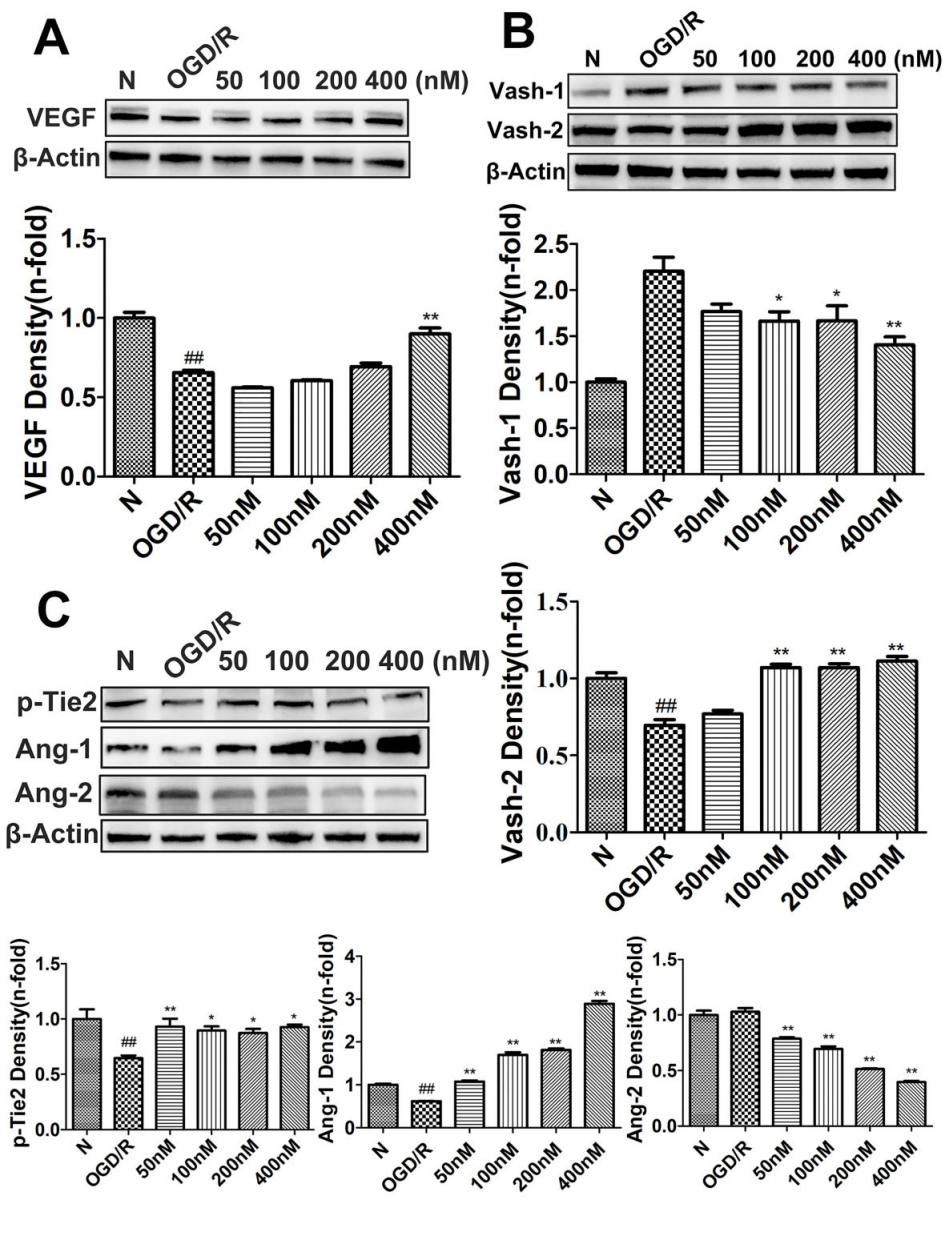
Human umbilical vein endothelial cells (HUVECs) were subjected to oxygen-glucose deprivation and reoxygenation (OGD/R) for 4 h prior to engeletin treatment for 24 h, after which western blotting was used to assess protein levels. **A** and **B**, Angiogenesis-related protein levels (VEGF, Vash-1, Vash-2). **C**, Vascular stability-related protein levels (Ang-1, Ang-2, *p*-Tie2). Data are reported as means±SD (n=3). *P<0.05, **P<0.01 *vs* OGD/R group; ^##^P<0.01 *vs* normal (N) group (ANOVA with Tukey's test). Vash: vasohibin.

**Figure 7:**
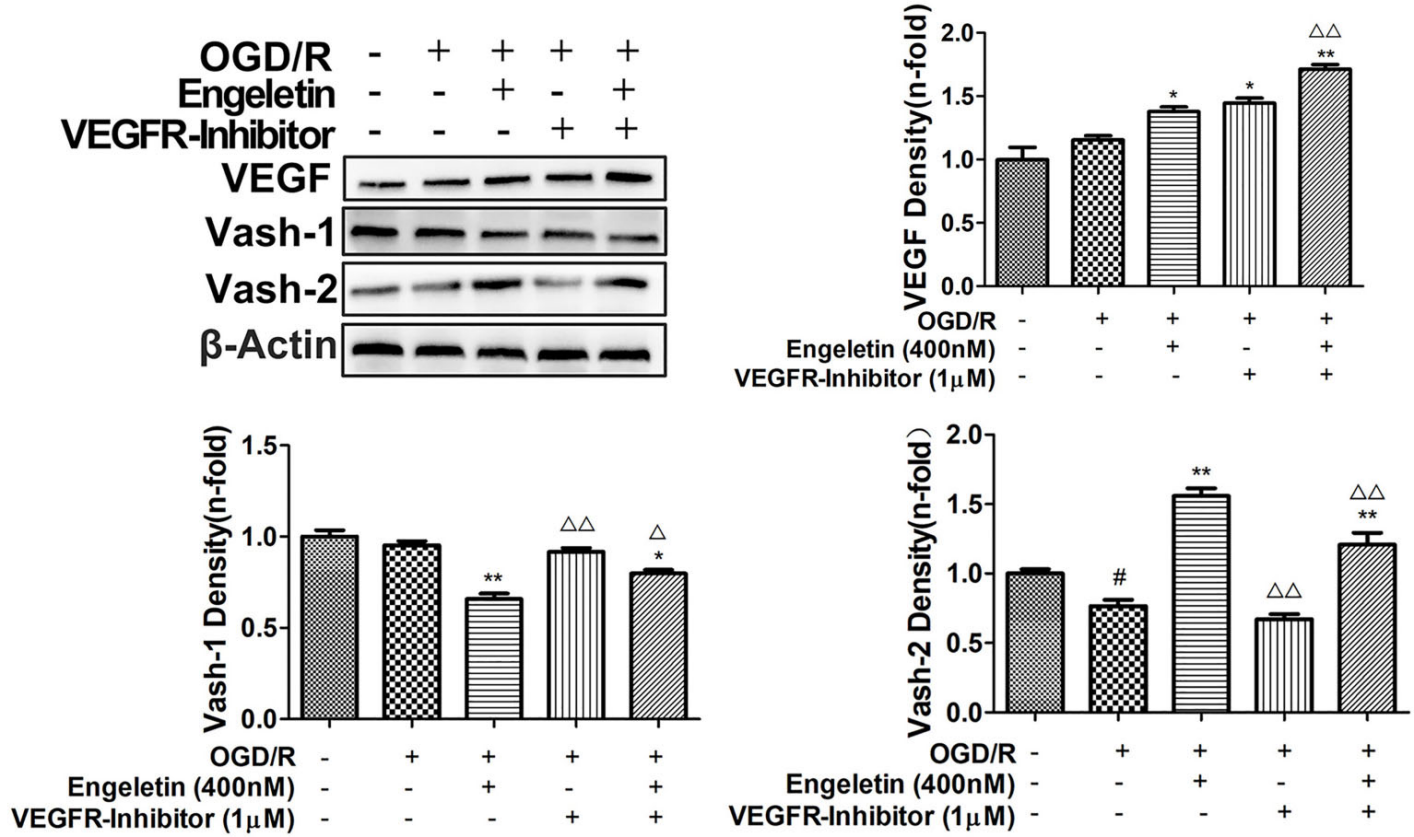
Human umbilical vein endothelial cells (HUVECs) were subjected to oxygen-glucose deprivation and reoxygenation (OGD/R) for 4 h prior to engeletin treatment for 24 h, after which western blotting was used to assess protein levels. Cells were treated with axitinib (1 μM, VEGFR-inhibitor) and engeletin (400 nM) for 24 h and then VEGF, Vash-1, and Vash-2 levels were assessed. Data are reported as means±SD (n=3). *P<0.05, **P<0.01 *vs* OGD/R group; ^#^P<0.05 *vs* control group. ^△^P<0.05, ^△△^P<0.01 *vs* engeletin group (ANOVA with Tukey's test). Vash: vasohibin.

To understand the mechanisms whereby engeletin promotes angiogenesis, we next evaluated the expression of key proteins related to this pathway. Levels of Vash-1 (F=9.798) and Ang-2 (F=16.881) were enhanced relative to sham group animals (Figures 2 and 5; P<0.05 or P<0.01). Western blotting revealed that treatment with engeletin (20 mg/kg) was sufficient to enhance the expression of Ang-1 (F=14.701), *p*-Tie2 (F=12.703), Vash-2 (F=63.007), and VEGF (F=43.526), relative to the MCAO group (P<0.05 or P<0.01). *In vitro* analyses yielded results comparable to those of our *in vivo* experiments (Figures 6 and 7). Following engeletin treatment (50-400 nM), HUVECs exhibited markedly enhanced VEGF (F=39.537), Vash-2 (F=50.526), Ang-1, and *p*-Tie2 expression in a dose-dependent fashion (P<0.05 or P<0.01). Engeletin thus exhibited neuroprotective activity in the context of CI/R via promoting angiogenesis through mechanisms that may be associated with the VEGF/vasohibin and Ang-1/Tie-2 pathways.

To further extend these results, we treated cells and rats with axitinib to block VEGF receptor activation. Axitinib treatment (10 mg/kg) was sufficient to reduce VEGF and Vash-2 expression relative to engeletin treatment alone (F=16.446, P<0.01; Figure 5), whereas Vash-1 levels were increased in response to axitinib treatment relative to engeletin treatment alone (F=27.992, P<0.01). Similarly, engeletin-induced increases in CD31, VEGF, and Vash-2 expression were significantly suppressed by axitinib (Figures 1 and 5), which had no impact on the Ang-1/Tie-2 pathway (Figure 2). Our *in vitro* findings were comparable to these *in vivo* results, with the inhibitory effects of axitinib (1 μM) on Vash-2 expression being particularly obvious in HUVECs (F=179.962, P<0.01, Figure 7).

## Discussion

VEGF is the primary endogenous driver of angiogenesis (25), promoting vascular endothelial cell migration, proliferation, and permeability. Vash-1 suppresses angiogenesis induction downstream of VEGF signaling via protein kinase C (PKC) (26). There is also evidence that Vash-1 suppresses the growth of tumors (27). Vash-2, in contrast, enhances intratumoral angiogenesis, and its function in the context of cerebral ischemia injury remains to be clarified (28). The VEGF/vasohibin signaling pathway may play a key role in a range of diseases owing to its ability to influence angiogenic signaling. As such, we measured VEGF, Vash-1, and Vash-2 protein levels in rats treated with engeletin and in HUVECs under similar experimental conditions. In all cases, engeletin promoted the expression of VEGF and Vash-2, whereas it suppressed Vash-1 expression relative to corresponding model groups. To confirm this mechanistic relationship, we additionally utilized the VEGF inhibitor axitinib, which markedly attenuated the beneficial effects of engeletin. Specifically, axitinib treatment was sufficient to suppress VEGF and Vash-2 expression and to enhance Vash-1 treatment. Overall, these data suggested that engeletin was able to promote VEGF and Vash-2 expression and to suppress Vash-1 expression in a dose-dependent manner. CD31 is a key endothelial cell marker (29), which was significantly upregulated after treatment with engeletin in MCAO model rats for 7 consecutive days, with VEGF and NeuN expression also being elevated following such treatment. However, these effects were reversed by axitinib treatment.

The promotion of angiogenesis is subsequently followed by differentiation and maturation (30), with VEGF being able to simultaneously induce vascular growth and permeability (31). Ang-1 is an oligomeric glycoprotein that binds to and signals through Tie-2 (32), thereby enhancing vascular stability and modulating associated permeability. Ang-1 binding to Tie-2 induces intracellular signaling that regulates endothelial cell survival, migration, and permeability (33). In contrast, Ang-2 functions as an antagonist that prevents Ang-1-induced Tie-2 phosphorylation, driving extensive angiogenesis and vascular destabilization (34). As such, we assessed Ang-1, Ang-2, and *p*-Tie2 levels in our experimental model systems. These analyses revealed that engeletin treatment significantly bolstered Ang-1 and *p*-Tie2 protein levels *in vitro* and *in vivo*, while simultaneously suppressing Ang-2 expression. We also found that axitinib did not impact the Ang-1/Tie-2 pathway *in vivo*. VEGF/vasohibin signaling is the primary driver of angiogenesis, while Ang-1/Tie-2 signaling is more important as a regulator of vascular maturation and stabilization (35). We confirmed that treatment with engeletin increased VEGF and Vash-2 expression and decreased Vash-1 expression in HUVECs and rats, while axitinib treatment reversed these effects. CD31, Ang-1, and *p*-Tie2 expression were also enhanced by engeletin, while Ang-2 was downregulated in response to such treatment. We therefore hypothesized that engeletin can mediate long-term protective angiogenesis in the context of CI/R injury via the VEGF/vasohibin and Ang-1/Tie-2 pathways.

Angiogenesis is a key mechanism that is central to long-term functional recovery following ischemic stroke (36). Endothelial cells migrate on an appropriate matrix and remodel into tubular structures, thereby forming an initial primitive vascular plexus (37). Herein, we assessed HUVEC migration through Transwell and wound healing assays and confirmed that engeletin treatment markedly enhanced the migratory abilities of these cells in both assays. These findings indicated that the VEGF/vasohibin and Ang-1/Tie-2 pathways mediated the enhancement of HUVEC migration and proliferation.

However, there are certain limitations to this study. First, we did not establish the pharmacokinetics-pharmacodynamics relationship for this treatment *in vivo* or *in vitro*. Second, at the tested engeletin concentrations and doses, several of the analyzed endpoints were not normalized to control levels following treatment. Third, CI/R injury is a complex, multi-faceted disease. In this study, we solely focused on the effects of engeletin on angiogenesis, and in future studies we will continue to explore whether engeletin can act on other pathways to play a protective role in the context of CI/R injury.

In conclusion, we found that engeletin treatment enhanced angiogenesis both *in vivo* and *in vitro*. Engeletin treatment preserved cerebral blood flow, neurological function, and survival *in vivo* through a mechanism that may be associated with VEGF/vasohibin signaling-mediated enhancement of angiogenesis and the stabilization of these new vessels via the Ang-1/Tie-2 pathway. Overall, our data provided important evidence regarding the mechanisms underlying the regulation of angiogenesis in this pathological context and provided an important theoretical foundation for utilizing engeletin to treat cerebral ischemia.
